# Antimicrobial susceptibility profile and molecular characterization of *Vibrio parahaemolyticus* strains isolated from imported shrimps

**DOI:** 10.1128/spectrum.00175-24

**Published:** 2024-06-04

**Authors:** Erwan Bourdonnais, Arnaud Briet, Thomas Brauge, Sabine Debuiche, Nicolas Helsens, Sophie A. Granier, Graziella Midelet

**Affiliations:** 1 French Agency for Food, Environmental and Occupational Health & Safety, Laboratory for Food Safety, Boulogne-sur-Mer, France; 2 French Agency for Food, Environmental and Occupational Health & Safety, Laboratory for Food Safety, Maisons-Alfort, France; University of Maryland Eastern Shore, Princess Anne, Maryland, USA

**Keywords:** *Vibrio parahaemolyticus*, antimicrobial resistance, phenotype, epidemiological cutoff, shrimps, whole-genome sequencing

## Abstract

*Vibrio parahaemolyticus* is a threat to human health and one of the leading bacterial causes of seafood-borne infection worldwide. This pathogen is autochtonous in the marine environment and is able to acquire antimicrobial resistance (AMR) mechanisms, which is a global concern. However, the emergence of AMR *V. parahaemolyticus* strains in seafood is still understudied, as interpretation criteria for this species for antimicrobial susceptibility tests are limited in the literature. In this study, we investigated the susceptibility profiles to clinically important antibiotics and the associated genetic determinants of *V. parahaemolyticus* isolates cultured from imported shrimps. Based on the analysis of the resistance phenotypes of 304 *V*. *parahaemolyticus* isolates, we have defined experimental epidemiological cutoff values (CO_WT_) for 14/15 antibiotics tested. We observed that 19.1% of the bacterial isolates had acquired resistance to at least one antibiotic class. The highest number of resistance was associated with tetracycline (14.5% of the strains) and trimethoprim-sulfamethoxazole (3.6%). Moreover, seven strains were multidrug-resistant (MDR, resistant to at least three antibiotic classes). The most frequently identified genes in these strains were *aph(3″)-Ib*/*aph(6)-Id* (aminoglycoside resistance), *sul2* (sulfonamide), *tet(59*) (tetracycline), and *floR* (chloramphenicol). The SXT/R391 family ICE and class 1 integron-integrase genes were detected by PCR in three and one MDR *V. parahaemolyticus* strains, respectively. Consequently, *V. parahaemolyticus* in seafood can act as a reservoir of AMR, constituting a health risk for the consumer.

**IMPORTANCE:**

Our study on “Antimicrobial Resistance Profiles and Genetic Determinants of *Vibrio parahaemolyticus* Isolates from Imported Shrimps” addresses a critical gap in understanding the emergence of antimicrobial resistance (AMR) in this seafood-associated pathogen. *Vibrio parahaemolyticus* is a major cause of global seafood-borne infections, and our research reveals that 19.1% of isolates from imported shrimps display resistance to at least one antibiotic class, with multidrug resistance observed in seven strains. Importantly, we establish experimental epidemiological cutoff values for antibiotic susceptibility, providing valuable criteria specific to *V. parahaemolyticus*. Our findings underscore the potential risk to consumers, emphasizing the need for vigilant monitoring and intervention strategies. This study significantly contributes to the comprehension of AMR dynamics in *V. parahaemolyticus*, offering crucial insights for global public health. The dissemination of our research through Microbiology Spectrum ensures broad accessibility and impact within the scientific community and beyond.

## INTRODUCTION

*Vibrio parahaemolyticus* is a Gram-negative bacterium naturally occurring in marine and estuarine environments. This bacterial species is a major food-borne pathogen that causes infections after the consumption of raw or undercooked seafood. Indeed, *V. parahaemolyticus* is frequently isolated from various seafoods such as finfish, oysters, clams, crabs, mussels, and shrimps worldwide (1). It is considered as an emergent risk in Europe and in the temperate seas due to global warming (2). Infection caused by *V. parahaemolyticus* is generally self-limiting, with symptoms ranging from abdominal cramps, nausea, diarrhea, fever, headache, and chills to septicemia in patients with underlying diseases infection (3). Antibiotics, like third-generation cephalosporin, fluoroquinolones, or azithromycin, can be prescribed to treat severe infections but their misuse or abuse can lead to the emergence of resistant strains (4, 5).

Bacteria from the *Vibrio* genus including *V. parahaemolyticus* are known for their genome plasticity. They possess two chromosomes, chromosomic integrons, and they can harbor conjugative elements such as plasmids or integrative and conjugative elements (ICE), like the SXT/R391 ICE family (6, 7). These mobile genetic elements (MGEs) are involved in the propagation and transmission of antimicrobial resistance genes (ARGs) between bacteria as well as the emergence of multidrug-resistant (MDR) strains. Thus, MDR strains of *V. parahaemolyticus* have been isolated from seafoods such as Horse Mackerel, Pacific Mackerel, shrimps, and shellfish mostly in Asia (8–11). All of these characteristics seem to indicate that *V. parahaemolyticus* may be a reservoir of ARGs and an actor of their dissemination between aquatic and human bacterial flora.

Nevertheless, identifying acquired antimicrobial resistance in *V. parahaemolyticus* is a challenge. To study the susceptibility of *V. parahaemolyticus* to antimicrobial compounds, the disk diffusion method is usually used. Clinical breakpoints had been defined for a clinical approach, between an antibiotic, a bacterium, and a host. It predicts the probability of success for an antibiotic therapy. According to EUCAST, bacteria are classified into four categories thanks to clinical breakpoints: (i) susceptible (S): there is a great chance of therapeutic success at standard dosing regimen; (ii) susceptible at increased exposure (I) : “when there is a high likelihood of therapeutic success because exposure to the agent is increased by adjusting the dosing regimen or by its concentration at the site of infection”; (iii) area of technical uncertainty (ATU) therapeutic success is uncertain; and (iv) resistant (R): there is a great chance of therapeutic failure. Clinical breakpoints do not suit to detect emergence of resistance and all resistance mechanisms. To detect acquired resistance in bacterial populations for epidemiological studies, the European Committee on Antimicrobial Susceptibility Testing (EUCAST) had defined epidemiological cutoff (ECOFF) values. ECOFF values allow setting apart the wild type (bacteria without acquired resistance mechanisms) and the non-wild type (bacteria with acquired resistance mechanisms to an antibiotic) populations. However, neither the CLSI, nor the EUCAST set ECOFF values for *V. parahaemolyticus* at the time of this study.

The aim of this study was to characterize acquired antimicrobial resistance in a collection of 304 *V*. *parahaemolyticus* strains isolated from shrimps imported to France. First, we tested 15 antibiotics of different classes critical for human health (β-lactams, quinolones, aminoglycosides, macrolides, phenicols, tetracyclines, and folate pathway inhibitors) by the disc diffusion method (12). We were thus able to determine experimental epidemiological cutoff (CO_WT_) values specific to the *V. parahaemolyticus* species for these antibiotics (13). Then, antimicrobial resistance mechanisms and markers of MGEs were investigated for the MDR *V. parahaemolyticus* isolates by PCR and complete genome sequencing.

## RESULTS

### Phenotypic analysis of antimicrobial resistance in *V. parahaemolyticus*

**Fig 1:**
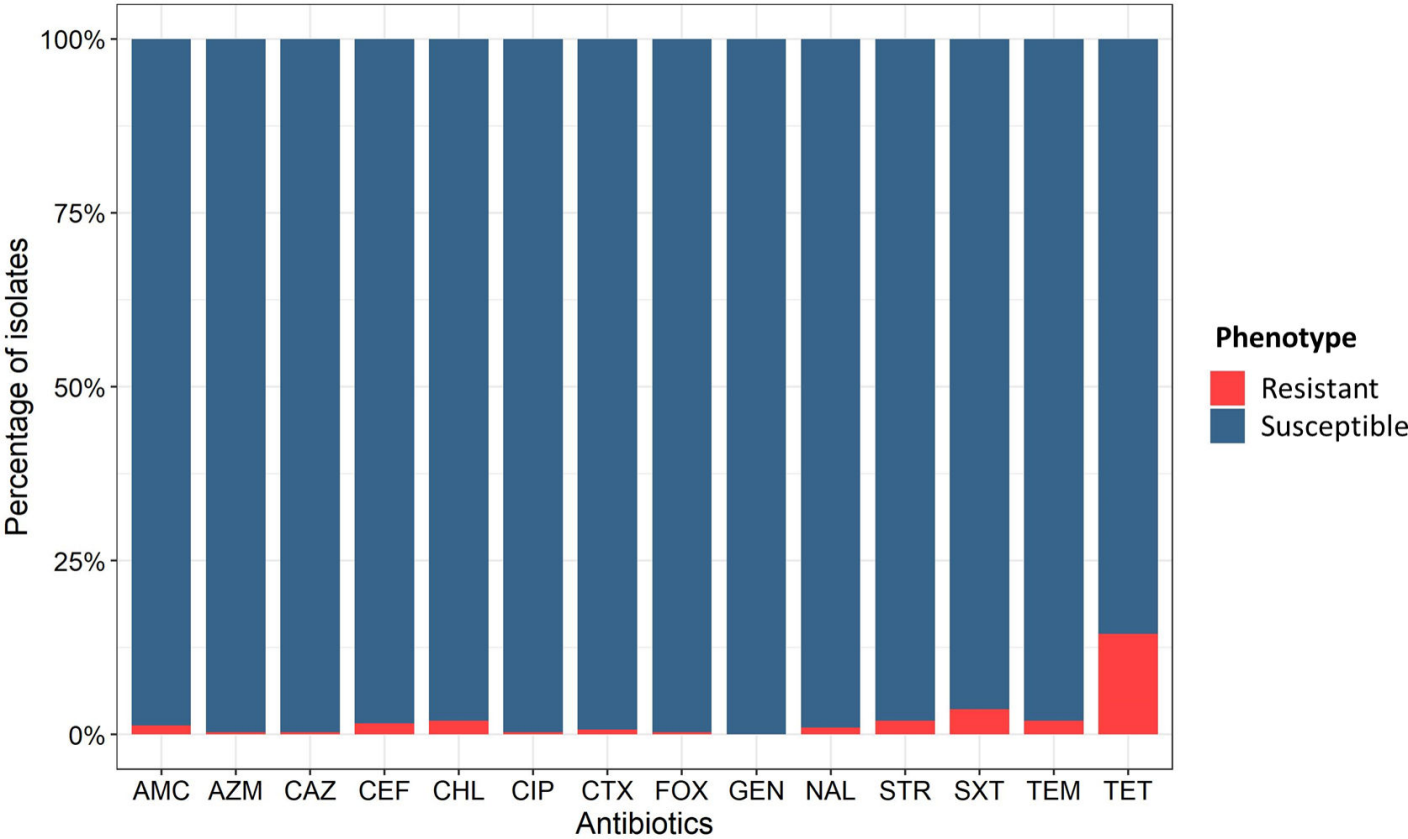
Antibiotic resistance pattern of the *V. parahaemolyticus* isolates (*n* = 304). AMC: amoxicillin-clavulanic acid; AZM: azithromycin; CAZ: ceftazidime; CEF: cephalothin; CHL: chloramphenicol; CIP: ciprofloxacin; CTX: cefotaxime; FOX: cefoxitin; GEN: gentamicin; NAL: nalidix acid; STR: streptomycin; SXT: trimethoprim-sulfamethoxazole; TEM: temocillin; TET: tetracycline.

Inhibition diameters for the 304 *V*. *parahaemolyticus* strains isolated from imported shrimps were determined for 15 antibiotics considered critical for human health (Fig. S1). Using the method described by Kronvall et al. (14), we calculated experimental epidemiological cutoff (CO_WT_) values for 14/15 antibiotics tested (Table 1). For ampicillin, the distribution of the inhibition zone diameter did not follow a normal law so it was impossible to determine a CO_WT_ value. For 14 other antibiotics tested, distribution followed a normal law and CO_WT_ values had been calculated. Using these CO_WT_ values, we were able to differentiate the wild-type (WT) population from the non-wild-type (non-WT) population that had acquired antibiotic resistance (Fig. 1). All antibiotics combined (excluding ampicillin), the non-WT population of *V. parahaemolyticus* represented 19.1% of the strains analyzed and 100% of the strains had a gentamicin-susceptible phenotype. The antibiotics for which the most isolates had a non-WT phenotype were tetracycline (14.5%), trimethoprim-sulfamethoxazole (3.6%), followed by chloramphenicol (2.0%), streptomycin (2.0%), and temocillin (2.0%). For the other antibiotics tested, we observed less than 2.0% of non-WT population. Among the non-WT isolates of *V. parahaemolyticus*, 14.5% have acquired resistance to one antibiotic and 2.0% to two antibiotics, with 4/6 of these isolates exhibiting a resistance phenotype to tetracycline and trimethoprim-sulfamethoxazole. We observed that seven isolates (2.3%) of *V. parahaemolyticus* were MDR, that is, resistant to at least three antibiotics. Indeed, 0.3% of the isolates had phenotypic resistance to three antibiotics and 1.6% to four antibiotics. Of these, 4/5 isolates were resistant to tetracycline, trimethoprim-sulfamethoxazole, streptomycin, and chloramphenicol. Finally, a *V. parahaemolyticus* MDR strain (16-B3PA-0006) exhibited resistance to nine antibiotics, whose phenotypic and genetic characterization has previously been described (15).

**TABLE 1:** Experimental epidemiological cutoff (CO_WT_) values and non-wild-type (non-WT) populations of *V. parahaemolyticus* (*n* = 304)^*a*^

Antibiotic class	Antibiotic	CO_WT_ (mm) WT≥	Number of non-WT isolates
β-lactams	Ampicillin	ND	ND
	Temocillin	25	6
	Amoxicillin-clavulanic acid	19	4
	Cephalothin	15	5
	Cefoxitin	16	1
	Ceftazidime	23	1
	Cefotaxime	27	2
Quinolones	Nalidixic acid	22	3
	Ciprofloxacin	20	1
Aminoglycosides	Streptomycin	10	6
	Gentamicin	14	0
Macrolides	Azithromycin	19	1
Phenicols	Chloramphenicol	26	6
Tetracyclines	Tetracycline	22	44
Folate pathway inhibitors	Trimethoprim-sulfamethoxazole	19	11

^*a*^ ND: Not determined.

### Genetic analysis of the MDR *V. parahaemolyticus* isolates

In order to identify the ARGs responsible for the observed phenotypic resistances, we sequenced the total genomes of the seven MDR isolates of *V. parahaemolyticus*. The results are shown in Table 2, including for the 16-B3PA-0006 isolate whose data has been previously published (15). The two *aph(3″)-Ib* (or “*strA*”) and *aph (6)-Id* (or “*strB*”) genes encoding aminoglycoside resistance were identified in the six MDR *V. parahaemolyticus* isolates exhibiting phenotypic resistance to streptomycin. These isolates, which were also resistant to trimethoprim-sulfamethoxazole, possessed the *sul2* gene (sulfonamide resistance). The *tet(59*) gene responsible for tetracycline resistance has been identified in five MDR isolates with phenotypic resistance to tetracycline and was associated with the *tetA* gene, which also codes for a tetracycline efflux pump, in the 13-B3PA-2931 isolate. For 5/7 MDR isolates, the *floR* gene encoding a chloramphenicol exporter was associated with phenotypic resistance to this antibiotic. The *bla*_CARB-26_, *bla*_CARB-31_, and *bla*_CARB-41_ genes coding for β-lactamases were identified in the 12-B3PA-1629, 16-B3PA-0166, 13-B3PA-2931,and 16-B3PA-0263 isolates. For the 12-B3PA-1629 isolate, no ARGs encoding resistance to tetracycline and chloramphenicol were identified despite phenotypic resistances to these two antibiotics. Furthermore, the *qnrA5* gene coding for a plasmid-mediated quinolone resistance was found in this strain although there was no phenotypic resistance to this antibiotic, and the *bla*_OXA-SHE_ gene (β-lactamase) was identified, which may explain the resistance to cephalothin. Finally, no nalidixic acid resistance gene was found in the 16-B3PA-0263 isolate despite the phenotypic resistance associated with this antibiotic. To determine the genetic support of these ARGs, we used the PlasmidFinder database, and performed PCRs targeting integron-integrase genes (classes 1, 2, and 3) and the SXT integrase. No plasmid were identified and all MDR *V. parahaemolyticus* isolates were negative for class 2 and 3 integron-integrases. Only the 16-B3PA-0006 isolate was positive for the class 1 integron-integrase. In addition, 3/7 isolates were positive for the SXT integrase.

**TABLE 2:** Antimicrobial resistance genes and integrases identified in the MDR *V. parahaemolyticus* isolates (*n* = 7)^*a*^

Isolate	Phenotypic resistances	Antimicrobial resistance genes	Integron/ICE
12-B3PA-1629	TET - CEF - CHL	*bla*_OXA-SHE_*bla*_CARB-31_ *qnrA5*	-
16-B3PA-0443	TET - STX - STR - CHL	*aph(3″)-Ib aph (6)-Id tet(59) sul2 floR*	SXT *int*
16-B3PA-0166	TET - STX - STR - CHL	*aph(3″)-Ib aph (6)-Id tet(59) sul2 floR bla*_CARB-41_	SXT *int*
15-B3PA-1773	TET - STX - STR - CHL	*aph(3″)-Ib aph (6)-Id tet(59) sul2 floR*	-
13-B3PA-2931	TET - STX - STR - CHL	*aph(3″)-Ib aph (6)-Id tet(59) tetA sul2 floR bla*_CARB-26_	-
16-B3PA-0263	TET - STX - STR - NAL	*aph(3″)-Ib aph (6)-Id tet(59) sul2 bla*_CARB-41_	SXT *int*
16-B3PA-0006^*b*^	AMC - CAZ - FOX - CTX - CEF - TET - STX - STR - CHL	*aph(3″)-Ib aph (6)-Id aadA2 tetA sul1 sul2 floR bla*_NDM-1_	*intI1*

^*a*^ AMC: amoxicillin-clavulanic acid; CAZ: ceftazidime; CEF: cephalothin; CHL: chloramphenicol; CTX: cefotaxime; FOX: cefoxitin; NAL: nalidix acid; STR: streptomycin; SXT: trimethoprim-sulfamethoxazole; TET: tetracycline.

^*b*^ Data previously published (15).

## DISCUSSION

In this study, we attempted to establish *V. parahaemolyticus* specific experimental cutoff values for 15 antibiotics in order to investigate acquired antimicrobial resistance in this bacterial species. By analyzing 304 *V*. *parahaemolyticus* isolates from imported shrimps, we calculated CO_WT_ values for 14/15 antibiotic molecules tested and non-wild-type population had been split from wild-type population *in vitro*. We observed that 80.9% of the isolates got a wild-type phenotype although acquired resistances were detected for tetracyclines in 14.5% of the isolates and for trimethoprim-sulfamethoxazole in 3.6% of the isolates. Furthermore, 2.0% of the isolates exhibited acquired resistance to chloramphenicol, streptomycin, and temocillin. *V. parahaemolyticus* strains with acquired resistance to these antibiotic classes had been described in coastal waters and sediment in the United States (16), short mackerel in Malaysia (17), sea cucumber in China (18), sea turtle in Mexico (19), and raw seafood in Poland (20). Resistance rates were variable depending on the matrix studied and the geographical area: from 0% to 17% for tetracyclines, 0.3% to 15% for folate pathway inhibitors, 75% to 100% for β-lactams, 3% to 46% for aminoglycosides, 0% to 12% for phenicols, and 0% to 3% for quinolones. Regarding macrolides, erythromycin was tested against *V. parahaemolyticus* in these studies but no breakpoint for this molecule is defined in the CLSI M45 document, so interpretation was not reliable. For tetracyclines, folate pathway inhibitors, phenicols, and quinolones, resistance rates in this study were in the range of the previous studies cited. It suggests that *V. parahaemolyticus* is able to acquire resistance mechanisms in various environments and can play a role in the antibiotic resistance spreading.

In the case of β-lactams, it was impossible to detect a non-WT population for ampicillin. Many studies have shown high rates of resistance in *V. parahaemolyticus* to penicillins such as penicillin G and ampicillin (16–20). Moreover, in this study, *bla*_CARB_ variants (encoding β-lactam resistance) were identified in 50% of the MDR *V. parahaemolyticus* isolates analyzed. However, its location on a genetic mobile element or on chromosomes could not be determined. Nevertheless, the inability to determine CO_WT_, the high resistance rate when the clinical breakpoint is used and the chromosomal carbenicillinase indicate an intrinsic resistance of *V. parahaemolyticus* toward penicillins (5). These molecules are not relevant to test for this bacterial species. More studies are needed to understand the impact of *bla*_CARB-17_ variant presence and expression in *V. parahaemolyticus* genomes on their natural resistance phenotype to some penicillins. If, as Chiou et al. suggested, *bla*_CARB-17_ gene mediates intrinsic resistance to penicillins in all *V. parahaemolyticus*, further investigations of the genomes apparently negative for *bla*_CARB_ in this study must be further explored.

It was noteworthy that we used streptomycin and gentamicin for testing resistance to aminoglycosides. Indeed, for streptomycin, clinical breakpoints were set at 15 mm for S/I and 11 mm for I/R according to the CLSI standards (21). In this study, the CO_WT_ calculated was 9 mm. It implies that part of the wild-type population in our study was classified as intermediate or resistant according to clinical breakpoints. It could lead to an overestimation of the number of *V. parahaemolyticus* strains with acquired resistance to aminoglycosides, like in the study of Tan et al. (17). They showed that 53% of *V. parahaemolyticus* strains were classified as clinically resistant to streptomycin. That is why, in order to detect a bacterial population with acquired resistance to antibiotics and not just clinically resistant bacteria, it is important to use epidemiological cutoff values even if they had to be set up experimentally. It also should avoid misinterpretation due to the use and extrapolation of interpretative criteria established from other bacterial genus/species. However, other studies are needed to establish ECOFF specific to *V. parahaemolyticus*. These ECOFFs will facilitate the detection of AMR *V. parahaemolyticus* strains in order to explore the diversity of ARGs and their genetic support.

To identify the ARGs involved in the phenotypic resistance observed in the MDR strains of *V. parahaemolyticus*, we sequenced their complete genome. As a reminder, the resistance genes for the 16-B3PA-0006 strain have already been identified (15). For the majority of these strains, we were able to associate ARGs with the phenotypic resistances. Thus, we have identified *tetA* and *tet(59*) (tetracyclines resistance), *aph(3″)-Ib* and *aph (6)-Id* (aminoglycosides resistance), *sul2* (sulfonamides resistance), *floR* (phenicols resistance), and *bla*_CARB-26_, *bla*_CARB-31_, and *bla*_CARB-41_ (β-lactam resistance). The identification of these ARGs was consistent with previous studies carried out on *V. parahaemolyticus* strains isolated from seafoods such as shrimps and sea cucumbers in Asia (18, 22–24). In this study, however, we observed discordances between resistance phenotypes and genotypes for two strains (12-B3PA-1629 and 16-B3PA-0263). For the first strain, the *qnr*A5 gene encoding a plasmid-mediated quinolone resistance was identified, but the phenotype associated was not observed. The inhibition diameters for nalidixic acid and ciprofloxacin were 4 mm above the CO_WT_ calculated, so these diameter measures were not in the grey zone of the measurement inaccuracy of 2 mm inherent of the diffusion method (25). Similarly, Poirel et al. (26) highlighted that the presence of the *qnrA* gene did not confer phenotypic resistance to quinolones (ciprofloxacin, ofloxacin, sparfloxacin, and norfloxacin) in *Shewanella algae* strains. This gene was associated with *bla*_OXA-SHE_, a gene coding for a class D β-lactamase (oxacillinase). The *bla*_OXA-SHE_ gene is 99% similar to *bla*_OXA-55_ and has been identified in clinical strains of *S. algae*, as well as in environmental strains isolated from red algae, wild invertebrates and fish from the Baltic Sea and in Japan (27). Similar to the *V. parahaemolyticus* 12-B3PA-1629 strain, the authors identified *qnrA*-type genes (A1, A2, A3, A5, and A7) associated with the *bla*_OXA-SHE_ gene. However, to our knowledge, this gene has never been identified in *V. parahaemolyticus*. For the 16-B3PA-0263 strain, the inhibition diameter for nalidixic acid and ciprofloxacin was 4 mm under the CO_WT_ so out of the measurement inaccuracy. Quinolone resistance can also be acquired by a mutation in the *gyr* or *par* genes, and it is probably the reason of the quinolone resistance in this strain. Finally, in the 12-B3PA-1629 strain, we did not identif ARGs associated with the tetracyclines and phenicols non-WT phenotype. As for the 16-B3PA-0263 strain and quinolone resistance, it might be a mutation or an unknown resistance mechanism. However, the diameter measure was 1 mm below the chloramphenicol CO_WT_, so it could be a false resistant strain. Hence, regarding antimicrobial resistance epidemiology, both resistance phenotypes and genotypes should be studied.

The diversity of resistance mechanisms described in *V. parahaemolyticus* suggests an ability for this species to cumulate ARGs, especially through the transfer of mobile genetic elements. Nevertheless, no plasmids were identified in the MDR *V. parahaemolyticus* isolates in our study. Mobile genetic structures such as ICEs also play a role in the acquisition of ARGs in *Vibrio*. In this study, three MDR isolates were positive for the SXT integrase. This ICE was rarely reported in *V. parahaemolyticus* strains and could carry ARGs: *tet*A*, str*A*, str*B*, flo*R*, sul*2*, dfr*A1, *and dfr*A18 (7, 18, 28, 29). However, a genetic comparison of ICEs SXT/R391 showed a variability in the integrase gene. This last gene is used as an epidemiological marker of this ICE family and its variability could lead to an underestimation of its prevalence in bacterial genomes, especially in *Vibrio* sp (7). The authors recommended a more conserved gene such as *set*CD to detect the presence of SXT/R391 ICEs. In contrast, integrons are poorly described in *V. parahaemolyticus*, even though they are used as environmental markers of antimicrobial resistance (30). In this study, one strain with acquired resistance was positive for a class 1 integron. Low prevalence of class 1 integrons and the absence of class 2 and 3 integrons in *V. parahaemolyticus* genomes had been also reported in other studies (28, 29, 31). In *V. parahaemolyticus,* it seems that antibiotic resistance is more frequently acquired through plasmids or ICEs than class 1, 2, and 3 integrons. Moreover, it has been demonstrated that *V. parahaemolyticus* is able to transfer plasmids carrying antimicrobial resistance genes to other *Vibrio* species, *Escherichia coli*, and other *Enterobacteriacae* (32).

This work has provided essential information for determining acquired antimicrobial resistance in *V. parahaemolyticus*, by setting species-specific experimental epidemiological cutoff values (CO_WT_). *V. parahaemolyticus*, in addition to being an emergent risk for food safety, might also play a role in antimicrobial resistance dissemination from marine environment to humans through seafood consumption. More studies are needed to assess the risk of antimicrobial resistance associated with *V. parahaemolyticus* to confirm the relevance of antimicrobial resistance monitoring in this bacterial species in imported seafood.

## MATERIALS AND METHODS

### *Vibrio parahaemolyticus* isolates

Between 2012 and 2016, 304 *V. parahaemolyticus* isolates were cultured from shrimps imported to France using the NF ISO/TS 21872-2007 standard (ANSES B3PA Collection). These strains were mostly from aquaculture and had different geographical origins, including from Ecuador (*n* = 54), Vietnam (*n* = 45), India (*n* = 17), Madagascar (*n* = 12), and Nigeria (*n* = 10) (Table S1). The isolates were stocked in brain heart infusion (Biomérieux, Lyon, France) with 20% (vol/vol) of glycerol at −80°C.

### Antimicrobial susceptibility test and experimental epidemiological cutoff (CO_wt_) values determination

All *V. parahaemolyticus* isolates were grown on saline nutritive agar (SNA, 2% NaCl) (Oxoid, Dardilly, France) at 37°C overnight. Antimicrobial susceptibility was performed by the disc diffusion method according to the instructions of the Clinical and Laboratory Standards Institute (21). A 0.5Mc Farland suspension was plated with a cotton swab on a Mueller-Hinton Agar (MHA) (Bio-Rad, Marne-la-Coquette, France) and the plates were incubated at 35°C for 16–18 h. The antibiotic disks tested were: ampicillin (10 µg), temocillin (30 µg), amoxicillin-clavulanic acid (20–10 µg), cephalothin (30 µg), cefoxitin (30 µg), ceftazidime (30 µg), cefotaxime (30 µg), nalidixic acid (30 µg), ciprofloxacin (5 µg), streptomycin (10 µg), gentamicin (10 µg), azithromycin (15 µg), chloramphenicol (30 µg), tetracycline (30 µg), and trimethoprim-sulfamethoxazole (1.25–23.75 µg) (Bio-Rad). A colisin disk (10 µg) was used to confirm strain identification and absence of contamination. Zone diameters were measured with a Biomic V3 (Giles Scientific, Santa Barbara, USA). The *E. coli* ATCC25922 strain was chosen as a quality control. Strains with a non-WT phenotype for at least three antibiotic classes were considered multidrug-resistant (MDR) (33).

Experimental epidemiological cutoff (CO_WT_) values were calculated using the method described by Kronvall et al. (14). The normalized resistance interpretation (NRI) method was applied with permission from the patent holder.

### PCR detection of the class 1, 2,and 3 integron-integrases and SXT/R391 ICE integrase

To perform the PCR reactions, DNA was extracted by simple cell lysis. For this purpose, few colonies of each MDR strain grown on SNA at 37°C overnight were suspended in 200 µL of Instagene Matrix (Bio-Rad). Suspension was incubated for 30 min at 56°C then for 8 min at 100°C. The tubes were centrifugated 5 min at 13,000 × *g* and the supernatant was used for the PCR reactions. The reaction mixture of 50 µL for the detection of the *intI1*, *intI2*, and *intI3* genes by PCR contained 5 µL of 10× PCR Buffer (Qiagen, Hilden, Germany), 0.2 mM of dNTPs (Eurobio, Les Ulis, France), 0.2 µM of each primer (Table 3) (Eurobio), 0.2 µL of Hot Start DNA Polymerase at 5 U.µL^−1^ (Qiagen), 2 µL of the supernatant containing *V. parahaemolyticus* DNA and nuclease-free water to complete the total reaction volume. For the detection of the SXT/R391 ICE integrase, 0.25 µM of each primer and 2.5 µL of the supernatant containing *V. parahaemolyticus* DNA were used. The *E. coli* DH5α/pTRC99A:*intI1*/*intI2*/*intI3* amp^R^ strain was used as a positive control for the detection of the *intI1*, *intI2*, and *intI3* genes (34). An *E. coli* strain containing the SXT ICE was used as a positive control for the SXT/R391 ICE integrase gene (Institut Pasteur, Paris). All PCR reactions were performed according to the conditions detailed in Table 4 using an iCycler thermal cycler (Bio-Rad).

**TABLE 3:** Primer information associated with the PCR reactions used in this study

Target gene	Primer name	Primer sequence (5′- > 3′)	Amplicon size (pb)	References
intI1	Int1F6	GCATCCTCGGTTTTCTGG	457	(35)
	Int1R6	GGTGTGGCGGGCTTCGTG		
intI2	Int2F	GTAGCAAACGAGTGACGAAATG	798	(36)
	Int2R	CACGGATATGCGACAAAAAGGT		
intI3	Int3R	GCCTCCGGCAGCGACTTTCAG	979	(36)
	Int3R	ACGGATCTGCCAAACCTGACT		
SXT int	SXT-F	GCTGGATAGGTTAAGGGCGG	592	(28)
	SXT-B	CTCTATGGGCACTGTCCACATTG		

**TABLE 4:** Experimental conditions associated with the PCR reactions used in this study

Target gene	PCR conditions
intI1	94°C —5 min (one cycle) ; 94°C —1 min, 55°C —1 min, 72°C —90 s (35 cycles) ; 72°C —10 min
intI2 and intI3	95°C —15 min (one cycle); 94°C —1 min, 59°C —1 min, 72°C —1 min (35 cycles) ; 72°C —10 min
SXT int	94°C —5 min (one cycle); 94°C —30 sec, 60°C —1 min, 72°C —2 min (35 cycles) ; 72°C —10 min

### Whole-genome sequencing of the MDR isolates

To perform the whole-genome sequencing, the genomic DNA of the MDR *V. parahaemolyticus* isolates was extracted from few colonies of the SNA with the DNeasy Blood & Tissue kit (Qiagen) following the manufacturer’s instructions for Gram-negative strains. DNA quality control and sequencing were carried out by Genoscreen Society (Lille, France). The antimicrobial resistance genes and the plasmids were identified with the ResFinder and PlasmidFinder softwares, respectively, from the CGE server (http://genomicepidemiology.org/ last access 30 May 2023). For the ResFinder database, the identity percentage threshold was set at 90% and the coverage percentage at 60%. For the PlasmidFinder database, the threshold values were set at 95% and 60%, respectively.

## SUPPLEMENTAL MATERIAL

Fig. S1 spectrum.00175-24-s0001.docx Distribution of inhibition diameters for 15 antibiotics.

Table S1 spectrum.00175-24-s0002.docx Summary of the 304 *V. parahaemolyticus* strains analyzed.

ASM does not own the copyrights to Supplemental Material that may be linked to, or accessed through, an article. The authors have granted ASM a non-exclusive, world-wide license to publish the Supplemental Material files. Please contact the corresponding author directly for reuse.
