# Supplementary material for: Antimicrobial susceptibility profile and molecular characterization of Vibrio parahaemolyticus strains isolated from imported shrimps
Source: Microbiol Spectr. 2024 Jun 4;12(7):e00175-24. doi: 10.1128/spectrum.00175-24 (PMC11218469; doi:10.1128/spectrum.00175-24)
Supplement: Fig. S1 — Distribution of inhibition diameters for 15 antibiotics. [file spectrum.00175-24-s0001.docx]

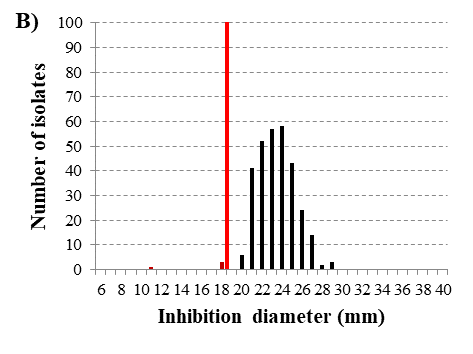

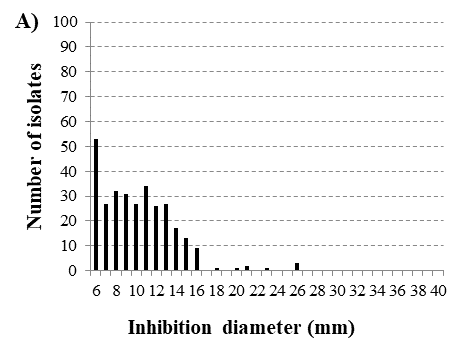

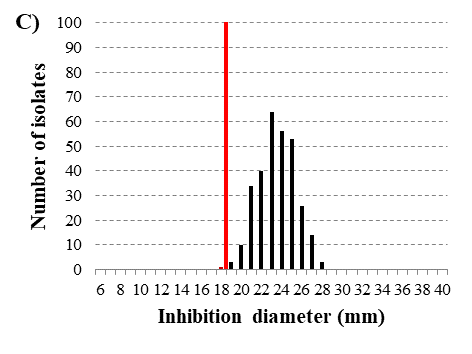

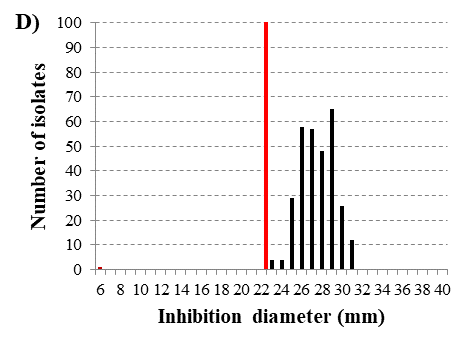

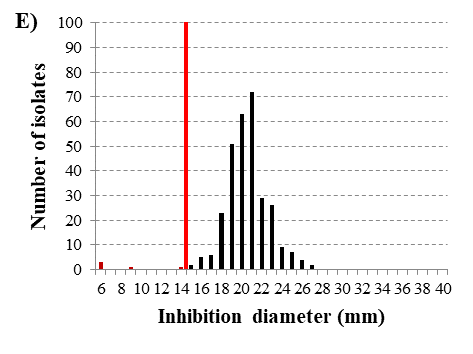

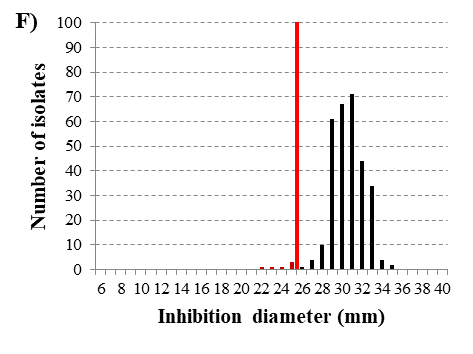


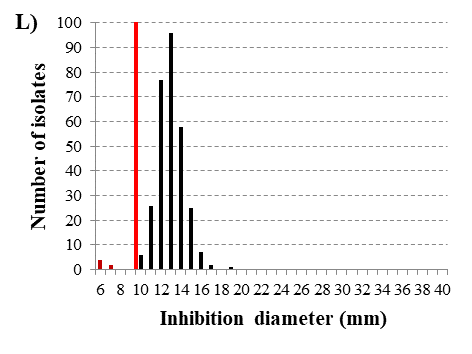

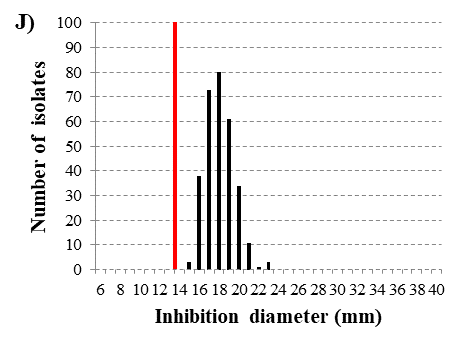

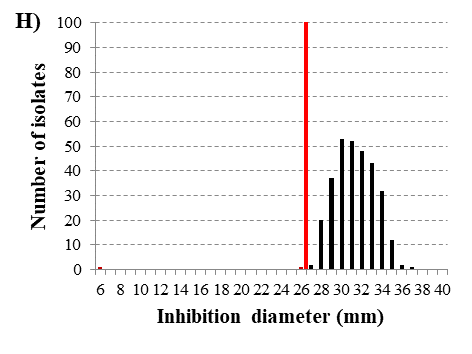

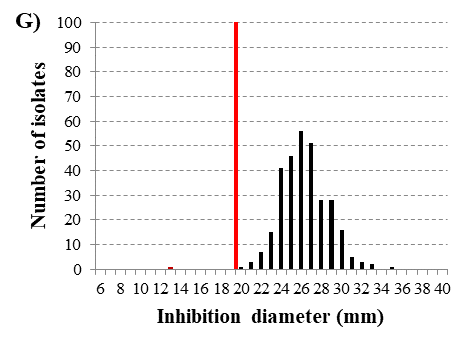

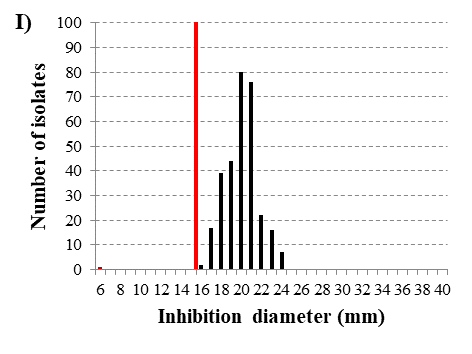

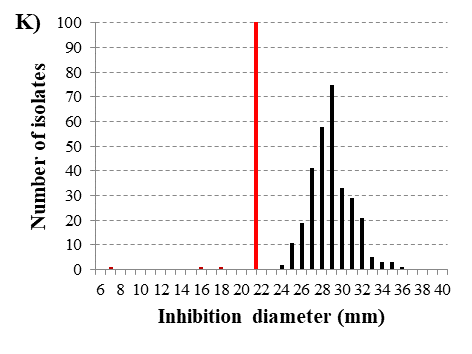


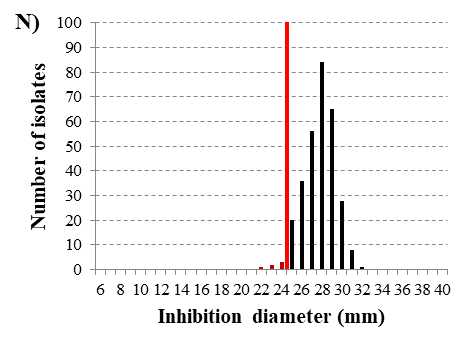

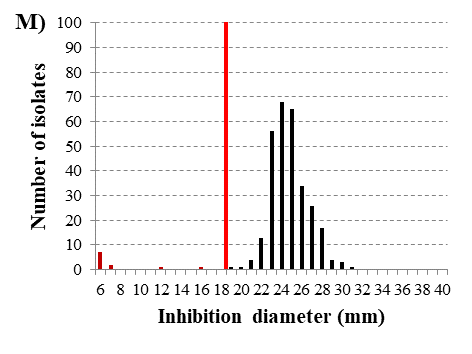

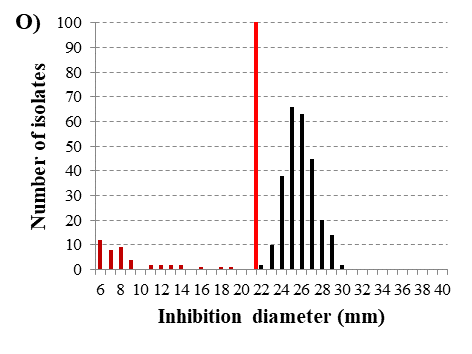


**Figure S1**: Distribution of inhibition diameters of the *V. parahaemolyticus* isolates (n=304) for 15 antibiotics. A) ampicillin. B) amoxicillin-clavulanic acid. C) azithromycin. D) ceftazidime. E) cephalothin. F) chloramphenicol. G) ciprofloxacin. H) cefotaxime. I) cefoxitin. J) gentamicin. K) nalidixic acid. L) streptomycin. M) trimethoprim-sulfamethoxazole. N) temocillin. O) tetracycline. The red bar indicates the experimental epidemiological cut-off (CO_WT_) value, the black histograms the wild type population (WT) and the red histograms the non-wild type population (non-WT).
