# Supplementary material for: Antimicrobial susceptibility profile and molecular characterization of Vibrio parahaemolyticus strains isolated from imported shrimps
Source: Microbiol Spectr. 2024 Jun 4;12(7):e00175-24. doi: 10.1128/spectrum.00175-24 (PMC11218469; doi:10.1128/spectrum.00175-24)
Supplement: Table S1 — Summary of the 304 V. parahaemolyticus strains analyzed. [file spectrum.00175-24-s0002.docx]

| Table S1: Summary of the 304 *V. parahaemolyticus* strains analysed in this study. | | | | |
| --- | --- | --- | --- | --- |
| **Strain** | **Species** | **Year** | **Food matrix** | **Geographical origin** |
| 12-B3PA-4660 | *V. parahaemolyticus* | 2012 | Shrimp | Eastern Central Atlantic |
| 12-B3PA-4661 | *V. parahaemolyticus* | 2012 | Shrimp | Eastern Central Atlantic |
| 12-B3PA-5760 | *V. parahaemolyticus* | 2012 | Shrimp | India |
| 12-B3PA-5943 | *V. parahaemolyticus* | 2012 | Shrimp | Vietnam |
| 12-B3PA-6026 | *V. parahaemolyticus* | 2012 | Shrimp | Madagascar |
| 12-B3PA-1628 J1 | *V. parahaemolyticus* | 2012 | Shrimp | Unknown |
| 12-B3PA-1629 J3 | *V. parahaemolyticus* | 2012 | Shrimp | Unknown |
| 12-B3PA-1629 J5 | *V. parahaemolyticus* | 2012 | Shrimp | Unknown |
| 12-B3PA-1825 | *V. parahaemolyticus* | 2012 | Shrimp | India |
| 12-B3PA-3209 V1 | *V. parahaemolyticus* | 2012 | Shrimp | Unknown |
| 12-B3PA-3941 V1 | *V. parahaemolyticus* | 2012 | Shrimp | Unknown |
| 12-B3PA-4399 J4 | *V. parahaemolyticus* | 2012 | Shrimp | Unknown |
| 12-B3PA-4399 J5 | *V. parahaemolyticus* | 2012 | Shrimp | Unknown |
| 12-B3PA-0790 J4 | *V. parahaemolyticus* | 2012 | Shrimp | Unknown |
| 12-B3PA-0790 J5 | *V. parahaemolyticus* | 2012 | Shrimp | Unknown |
| 13-B3PA-0187 | *V. parahaemolyticus* | 2013 | Shrimp | Ecuador |
| 13-B3PA-0440 | *V. parahaemolyticus* | 2013 | Shrimp | Ecuador |
| 13-B3PA-0614 | *V. parahaemolyticus* | 2013 | Shrimp | Unknown |
| 13-B3PA-0615 | *V. parahaemolyticus* | 2013 | Shrimp | Unknown |
| 13-B3PA-0617 | *V. parahaemolyticus* | 2013 | Shrimp | Unknown |
| 13-B3PA-0632 | *V. parahaemolyticus* | 2013 | Shrimp | Ecuador |
| 13-B3PA-0758 | *V. parahaemolyticus* | 2013 | Shrimp | Ecuador |
| 13-B3PA-1163 | *V. parahaemolyticus* | 2013 | Shrimp | Ecuador |
| 13-B3PA-1448 | *V. parahaemolyticus* | 2013 | Shrimp | Madagascar |
| 13-B3PA-1875 | *V. parahaemolyticus* | 2013 | Shrimp | Honduras |
| 13-B3PA-1876 | *V. parahaemolyticus* | 2013 | Shrimp | Honduras |
| 13-B3PA-1877 | *V. parahaemolyticus* | 2013 | Shrimp | India |
| 13-B3PA-1987 | *V. parahaemolyticus* | 2013 | Shrimp | Ecuador |
| 13-B3PA-2931 | *V. parahaemolyticus* | 2013 | Shrimp | Vietnam |
| 13-B3PA-3008 | *V. parahaemolyticus* | 2013 | Shrimp | India |
| 13-B3PA-3038 | *V. parahaemolyticus* | 2013 | Shrimp | Indian Ocean |
| 13-B3PA-3051 | *V. parahaemolyticus* | 2013 | Shrimp | Nigeria |
| 14-B3PA-0046 | *V. parahaemolyticus* | 2014 | Shrimp | Nigeria |
| 14-B3PA-0553 | *V. parahaemolyticus* | 2014 | Shrimp | Nicaragua |
| 14-B3PA-0608 | *V. parahaemolyticus* | 2014 | Shrimp | Thailand |
| 14-B3PA-0993 | *V. parahaemolyticus* | 2014 | Shrimp | Nigeria |
| 14-B3PA-1258 | *V. parahaemolyticus* | 2014 | Shrimp | Ecuador |
| 14-B3PA-1321 | *V. parahaemolyticus* | 2014 | Shrimp | India |
| 14-B3PA-1387 | *V. parahaemolyticus* | 2014 | Shrimp | Ecuador |
| 14-B3PA-1417 | *V. parahaemolyticus* | 2014 | Shrimp | India |
| 14-B3PA-1553 | *V. parahaemolyticus* | 2014 | Shrimp | Unknown |
| 14-B3PA-1864 | *V. parahaemolyticus* | 2014 | Shrimp | Ecuador |
| 14-B3PA-1936 | *V. parahaemolyticus* | 2014 | Shrimp | Honduras |
| 14-B3PA-1974 | *V. parahaemolyticus* | 2014 | Shrimp | Honduras |
| 14-B3PA-1975 | *V. parahaemolyticus* | 2014 | Shrimp | Madagascar |
| 14-B3PA-1976 | *V. parahaemolyticus* | 2014 | Shrimp | India |
| 14-B3PA-2359 | *V. parahaemolyticus* | 2014 | Shrimp | Madagascar |
| 14-B3PA-2360 | *V. parahaemolyticus* | 2014 | Shrimp | Central Atlantic |
| 14-B3PA-2420 | *V. parahaemolyticus* | 2014 | Shrimp | Unknown |
| 14-B3PA-3218 | *V. parahaemolyticus* | 2014 | Shrimp | Ecuador |
| 14-B3PA-3224 | *V. parahaemolyticus* | 2014 | Shrimp | Ecuador |
| 15-B3PA-0177 J3 | *V. parahaemolyticus* | 2015 | Shrimp | Vietnam |
| 15-B3PA-0709 J4 | *V. parahaemolyticus* | 2015 | Shrimp | Vietnam |
| 16-B3PA-0006 | *V. parahaemolyticus* | 2016 | Shrimp | Vietnam |
| 16-B3PA-0008 | *V. parahaemolyticus* | 2016 | Shrimp | Vietnam |
| 15-B3PA-0709 J2 | *V. parahaemolyticus* | 2015 | Shrimp | Vietnam |
| 15-B3PA-0709 M1 | *V. parahaemolyticus* | 2015 | Shrimp | Vietnam |
| 15-B3PA-0752 | *V. parahaemolyticus* | 2015 | Shrimp | India |
| 15-B3PA-0946 | *V. parahaemolyticus* | 2015 | Shrimp | Unknown |
| 15-B3PA-0954 | *V. parahaemolyticus* | 2015 | Shrimp | Unknown |
| 15-B3PA-0955 | *V. parahaemolyticus* | 2015 | Shrimp | Unknown |
| 15-B3PA-0956 | *V. parahaemolyticus* | 2015 | Shrimp | Unknown |
| 15-B3PA-0957 | *V. parahaemolyticus* | 2015 | Shrimp | Unknown |
| 15-B3PA-0958 | *V. parahaemolyticus* | 2015 | Shrimp | India |
| 15-B3PA-0996 | *V. parahaemolyticus* | 2015 | Shrimp | Unknown |
| 15-B3PA-0998 | *V. parahaemolyticus* | 2015 | Shrimp | Ecuador |
| 15-B3PA-0999 | *V. parahaemolyticus* | 2015 | Shrimp | Ecuador |
| 15-B3PA-0709 V1 | *V. parahaemolyticus* | 2015 | Shrimp | Vietnam |
| 15-B3PA-0709 V2 | *V. parahaemolyticus* | 2015 | Shrimp | Vietnam |
| 15-B3PA-1081 M1 | *V. parahaemolyticus* | 2015 | Shrimp | Vietnam |
| 15-B3PA-1081 M1.2 | *V. parahaemolyticus* | 2015 | Shrimp | Vietnam |
| 15-B3PA-1081 M2 | *V. parahaemolyticus* | 2015 | Shrimp | Vietnam |
| 15-B3PA-1081 M2.2 | *V. parahaemolyticus* | 2015 | Shrimp | Vietnam |
| 15-B3PA-1081 M3 | *V. parahaemolyticus* | 2015 | Shrimp | Vietnam |
| 15-B3PA-1081 M3.2 | *V. parahaemolyticus* | 2015 | Shrimp | Vietnam |
| 15-B3PA-1081 M4 | *V. parahaemolyticus* | 2015 | Shrimp | Vietnam |
| 15-B3PA-1081 M4.2 | *V. parahaemolyticus* | 2015 | Shrimp | Vietnam |
| 15-B3PA-1081 M5 | *V. parahaemolyticus* | 2015 | Shrimp | Vietnam |
| 15-B3PA-1081 M5.2 | *V. parahaemolyticus* | 2015 | Shrimp | Vietnam |
| 15-B3PA-1081 V1 | *V. parahaemolyticus* | 2015 | Shrimp | Vietnam |
| 15-B3PA-1081 V1.2 | *V. parahaemolyticus* | 2015 | Shrimp | Vietnam |
| 15-B3PA-1081 V1.3 | *V. parahaemolyticus* | 2015 | Shrimp | Vietnam |
| 15-B3PA-1081 V10 | *V. parahaemolyticus* | 2015 | Shrimp | Vietnam |
| 15-B3PA-1081 V2 | *V. parahaemolyticus* | 2015 | Shrimp | Vietnam |
| 15-B3PA-1081 V2.2 | *V. parahaemolyticus* | 2015 | Shrimp | Vietnam |
| 15-B3PA-1081 V3 | *V. parahaemolyticus* | 2015 | Shrimp | Vietnam |
| 15-B3PA-1081 V4 | *V. parahaemolyticus* | 2015 | Shrimp | Vietnam |
| 15-B3PA-1081 V4.2 | *V. parahaemolyticus* | 2015 | Shrimp | Vietnam |
| 15-B3PA-1081 V5 | *V. parahaemolyticus* | 2015 | Shrimp | Vietnam |
| 15-B3PA-1129 | *V. parahaemolyticus* | 2015 | Shrimp | Ecuador |
| 15-B3PA-1130 | *V. parahaemolyticus* | 2015 | Shrimp | Ecuador |
| 15-B3PA-1131 | *V. parahaemolyticus* | 2015 | Shrimp | Ecuador |
| 15-B3PA-1132 | *V. parahaemolyticus* | 2015 | Shrimp | Ecuador |
| 15-B3PA-1256 | *V. parahaemolyticus* | 2015 | Shrimp | Nigeria |
| 15-B3PA-1285 | *V. parahaemolyticus* | 2015 | Shrimp | Unknown |
| 15-B3PA-1291 | *V. parahaemolyticus* | 2015 | Shrimp | Unknown |
| 15-B3PA-1315 | *V. parahaemolyticus* | 2015 | Shrimp | Unknown |
| 15-B3PA-1316 | *V. parahaemolyticus* | 2015 | Shrimp | Unknown |
| 15-B3PA-1317 | *V. parahaemolyticus* | 2015 | Shrimp | Unknown |
| 15-B3PA-1349 | *V. parahaemolyticus* | 2015 | Shrimp | Unknown |
| 15-B3PA-1350 | *V. parahaemolyticus* | 2015 | Shrimp | Unknown |
| 15-B3PA-1357 | *V. parahaemolyticus* | 2015 | Shrimp | Madagascar |
| 15-B3PA-1386 | *V. parahaemolyticus* | 2015 | Shrimp | Unknown |
| 15-B3PA-1081 V5.2 | *V. parahaemolyticus* | 2015 | Shrimp | Vietnam |
| 15-B3PA-1412 | *V. parahaemolyticus* | 2015 | Shrimp | Unknown |
| 15-B3PA-1460 | *V. parahaemolyticus* | 2015 | Shrimp | Unknown |
| 15-B3PA-1521 | *V. parahaemolyticus* | 2015 | Shrimp | Unknown |
| 15-B3PA-1522 | *V. parahaemolyticus* | 2015 | Shrimp | Unknown |
| 15-B3PA-1546 | *V. parahaemolyticus* | 2015 | Shrimp | India |
| 15-B3PA-1552 | *V. parahaemolyticus* | 2015 | Shrimp | Ecuador |
| 15-B3PA-1554 | *V. parahaemolyticus* | 2015 | Shrimp | Ecuador |
| 15-B3PA-1556.2 | *V. parahaemolyticus* | 2015 | Shrimp | Nigeria |
| 15-B3PA-1581 | *V. parahaemolyticus* | 2015 | Shrimp | Nicaragua |
| 15-B3PA-1583 | *V. parahaemolyticus* | 2015 | Shrimp | Nicaragua |
| 15-B3PA-1591 | *V. parahaemolyticus* | 2015 | Shrimp | Honduras |
| 15-B3PA-1592 | *V. parahaemolyticus* | 2015 | Shrimp | Unknown |
| 15-B3PA-1598 | *V. parahaemolyticus* | 2015 | Shrimp | Unknown |
| 15-B3PA-1645 | *V. parahaemolyticus* | 2015 | Shrimp | Unknown |
| 15-B3PA-1651 | *V. parahaemolyticus* | 2015 | Shrimp | Unknown |
| 15-B3PA-1669 | *V. parahaemolyticus* | 2015 | Shrimp | Honduras |
| 15-B3PA-1720 | *V. parahaemolyticus* | 2015 | Shrimp | Nigeria |
| 15-B3PA-1751 | *V. parahaemolyticus* | 2015 | Shrimp | India |
| 15-B3PA-1767 | *V. parahaemolyticus* | 2015 | Shrimp | Unknown |
| 15-B3PA-1768 | *V. parahaemolyticus* | 2015 | Shrimp | Unknown |
| 15-B3PA-1769 | *V. parahaemolyticus* | 2015 | Shrimp | Nigeria |
| 15-B3PA-1770.2 | *V. parahaemolyticus* | 2015 | Shrimp | Unknown |
| 15-B3PA-1771 | *V. parahaemolyticus* | 2015 | Shrimp | Unknown |
| 15-B3PA-1772 | *V. parahaemolyticus* | 2015 | Shrimp | Unknown |
| 15-B3PA-1773 | *V. parahaemolyticus* | 2015 | Shrimp | Unknown |
| 15-B3PA-1891 | *V. parahaemolyticus* | 2015 | Shrimp | Ecuador |
| 15-B3PA-1932 | *V. parahaemolyticus* | 2015 | Shrimp | Madagascar |
| 15-B3PA-1965 | *V. parahaemolyticus* | 2015 | Shrimp | India |
| 15-B3PA-1966 | *V. parahaemolyticus* | 2015 | Shrimp | Unknown |
| 15-B3PA-1967 | *V. parahaemolyticus* | 2015 | Shrimp | India |
| 15-B3PA-1968 | *V. parahaemolyticus* | 2015 | Shrimp | Panama |
| 15-B3PA-1969 | *V. parahaemolyticus* | 2015 | Shrimp | Ecuador |
| 15-B3PA-1970 | *V. parahaemolyticus* | 2015 | Shrimp | Nigeria |
| 15-B3PA-2000 M2 | *V. parahaemolyticus* | 2015 | Shrimp | Unknown |
| 15-B3PA-2000 V1 | *V. parahaemolyticus* | 2015 | Shrimp | Unknown |
| 15-B3PA-2000 V1.2 | *V. parahaemolyticus* | 2015 | Shrimp | Unknown |
| 15-B3PA-2001 J1 | *V. parahaemolyticus* | 2015 | Shrimp | Unknown |
| 15-B3PA-2001 M1 | *V. parahaemolyticus* | 2015 | Shrimp | Unknown |
| 15-B3PA-2001 V1 | *V. parahaemolyticus* | 2015 | Shrimp | Unknown |
| 15-B3PA-2013 | *V. parahaemolyticus* | 2015 | Shrimp | Ecuador |
| 15-B3PA-2014 | *V. parahaemolyticus* | 2015 | Shrimp | Ecuador |
| 15-B3PA-2015 | *V. parahaemolyticus* | 2015 | Shrimp | Ecuador |
| 15-B3PA-2175 | *V. parahaemolyticus* | 2015 | Shrimp | Eastern Central Atlantic |
| 15-B3PA-2178 | *V. parahaemolyticus* | 2015 | Shrimp | Madagascar |
| 15-B3PA-1081 V9 | *V. parahaemolyticus* | 2015 | Shrimp | Vietnam |
| 15-B3PA-1406 | *V. parahaemolyticus* | 2015 | Shrimp | Vietnam |
| 16-B3PA-0007 | *V. parahaemolyticus* | 2016 | Shrimp | Vietnam |
| 16-B3PA-0036 | *V. parahaemolyticus* | 2016 | Shrimp | Ecuador |
| 16-B3PA-0112 | *V. parahaemolyticus* | 2016 | Shrimp | Unknown |
| 16-B3PA-0114 | *V. parahaemolyticus* | 2016 | Shrimp | Unknown |
| 16-B3PA-0115 | *V. parahaemolyticus* | 2016 | Shrimp | Unknown |
| 16-B3PA-0125 | *V. parahaemolyticus* | 2016 | Shrimp | Unknown |
| 16-B3PA-0155 WT | *V. parahaemolyticus* | 2016 | Shrimp | Unknown |
| 16-B3PA-0159 | *V. parahaemolyticus* | 2016 | Shrimp | Unknown |
| 16-B3PA-0160 | *V. parahaemolyticus* | 2016 | Shrimp | Unknown |
| 16-B3PA-0166 | *V. parahaemolyticus* | 2016 | Shrimp | Unknown |
| 16-B3PA-0168 | *V. parahaemolyticus* | 2016 | Shrimp | Unknown |
| 16-B3PA-0192 | *V. parahaemolyticus* | 2016 | Shrimp | Vietnam |
| 16-B3PA-0193 | *V. parahaemolyticus* | 2016 | Shrimp | Vietnam |
| 16-B3PA-0194 | *V. parahaemolyticus* | 2016 | Shrimp | Vietnam |
| 16-B3PA-0195 | *V. parahaemolyticus* | 2016 | Shrimp | Vietnam |
| 16-B3PA-0198 | *V. parahaemolyticus* | 2016 | Shrimp | Unknown |
| 16-B3PA-0200 | *V. parahaemolyticus* | 2016 | Shrimp | Honduras |
| 16-B3PA-0212 | *V. parahaemolyticus* | 2016 | Shrimp | Unknown |
| 16-B3PA-0213 | *V. parahaemolyticus* | 2016 | Shrimp | Unknown |
| 16-B3PA-0225 | *V. parahaemolyticus* | 2016 | Shrimp | Madagascar |
| 16-B3PA-0263 | *V. parahaemolyticus* | 2016 | Shrimp | Unknown |
| 16-B3PA-0266 | *V. parahaemolyticus* | 2016 | Shrimp | Unknown |
| 16-B3PA-0269 | *V. parahaemolyticus* | 2016 | Shrimp | Ecuador |
| 16-B3PA-0280 | *V. parahaemolyticus* | 2016 | Shrimp | Unknown |
| 16-B3PA-0285 | *V. parahaemolyticus* | 2016 | Shrimp | Unknown |
| 16-B3PA-0286 | *V. parahaemolyticus* | 2016 | Shrimp | Unknown |
| 16-B3PA-0408 | *V. parahaemolyticus* | 2016 | Shrimp | Unknown |
| 16-B3PA-0344 | *V. parahaemolyticus* | 2016 | Shrimp | Unknown |
| 16-B3PA-0408 | *V. parahaemolyticus* | 2016 | Shrimp | Unknown |
| 16-B3PA-0441 | *V. parahaemolyticus* | 2016 | Shrimp | Vietnam |
| 16-B3PA-0442 | *V. parahaemolyticus* | 2016 | Shrimp | Vietnam |
| 16-B3PA-0443 | *V. parahaemolyticus* | 2016 | Shrimp | Vietnam |
| 16-B3PA-0444 | *V. parahaemolyticus* | 2016 | Shrimp | Vietnam |
| 16-B3PA-0512 | *V. parahaemolyticus* | 2016 | Shrimp | Unknown |
| 16-B3PA-0526 | *V. parahaemolyticus* | 2016 | Shrimp | Unknown |
| 16-B3PA-0527 | *V. parahaemolyticus* | 2016 | Shrimp | Unknown |
| 16-B3PA-0540 | *V. parahaemolyticus* | 2016 | Shrimp | Unknown |
| 16-B3PA-0555 | *V. parahaemolyticus* | 2016 | Shrimp | Unknown |
| 16-B3PA-0556 | *V. parahaemolyticus* | 2016 | Shrimp | Unknown |
| 16-B3PA-0560 | *V. parahaemolyticus* | 2016 | Shrimp | Ecuador |
| 16-B3PA-0561 | *V. parahaemolyticus* | 2016 | Shrimp | Honduras |
| 16-B3PA-0566 | *V. parahaemolyticus* | 2016 | Shrimp | Nigeria |
| 16-B3PA-0879 | *V. parahaemolyticus* | 2016 | Shrimp | Unknown |
| 16-B3PA-0919 | *V. parahaemolyticus* | 2016 | Shrimp | Unknown |
| 16-B3PA-0920 | *V. parahaemolyticus* | 2016 | Shrimp | Vietnam |
| 16-B3PA-0925 | *V. parahaemolyticus* | 2016 | Shrimp | Unknown |
| 16-B3PA-0964 | *V. parahaemolyticus* | 2016 | Shrimp | Vietnam |
| 16-B3PA-0966 | *V. parahaemolyticus* | 2016 | Shrimp | Vietnam |
| 16-B3PA-1097 | *V. parahaemolyticus* | 2016 | Shrimp | Unknown |
| 16-B3PA-1373 | *V. parahaemolyticus* | 2016 | Shrimp | Unknown |
| 16-B3PA-1374 | *V. parahaemolyticus* | 2016 | Shrimp | Unknown |
| 16-B3PA-1375 | *V. parahaemolyticus* | 2016 | Shrimp | Unknown |
| 16-B3PA-1376 | *V. parahaemolyticus* | 2016 | Shrimp | Unknown |
| 16-B3PA-1377 | *V. parahaemolyticus* | 2016 | Shrimp | Unknown |
| 16-B3PA-1378 | *V. parahaemolyticus* | 2016 | Shrimp | Unknown |
| 16-B3PA-1430 | *V. parahaemolyticus* | 2016 | Shrimp | Unknown |
| 16-B3PA-1481 | *V. parahaemolyticus* | 2016 | Shrimp | Unknown |
| 16-B3PA-1482 | *V. parahaemolyticus* | 2016 | Shrimp | Unknown |
| 16-B3PA-1531 | *V. parahaemolyticus* | 2016 | Shrimp | Ecuador |
| 16-B3PA-1634 | *V. parahaemolyticus* | 2016 | Shrimp | Unknown |
| 16-B3PA-1742 | *V. parahaemolyticus* | 2016 | Shrimp | Unknown |
| 16-B3PA-2112 | *V. parahaemolyticus* | 2016 | Shrimp | Unknown |
| 16-B3PA-2144 | *V. parahaemolyticus* | 2016 | Shrimp | Madagascar |
| 16-B3PA-2145 | *V. parahaemolyticus* | 2016 | Shrimp | Madagascar |
| 16-B3PA-2146 | *V. parahaemolyticus* | 2016 | Shrimp | Unknown |
| 16-B3PA-2162 | *V. parahaemolyticus* | 2016 | Shrimp | Unknown |
| 16-B3PA-2163 | *V. parahaemolyticus* | 2016 | Shrimp | Unknown |
| 16-B3PA-2164 | *V. parahaemolyticus* | 2016 | Shrimp | Unknown |
| 16-B3PA-2165 | *V. parahaemolyticus* | 2016 | Shrimp | Unknown |
| 16-B3PA-2166 | *V. parahaemolyticus* | 2016 | Shrimp | Unknown |
| 16-B3PA-2168 | *V. parahaemolyticus* | 2016 | Shrimp | Unknown |
| 16-B3PA-2169 | *V. parahaemolyticus* | 2016 | Shrimp | Unknown |
| 16-B3PA-2182 | *V. parahaemolyticus* | 2016 | Shrimp | Ecuador |
| 16-B3PA-2183 | *V. parahaemolyticus* | 2016 | Shrimp | Ecuador |
| 16-B3PA-2202 | *V. parahaemolyticus* | 2016 | Shrimp | Unknown |
| 16-B3PA-2204 | *V. parahaemolyticus* | 2016 | Shrimp | Unknown |
| 16-B3PA-2280 | *V. parahaemolyticus* | 2016 | Shrimp | Unknown |
| 16-B3PA-2393 | *V. parahaemolyticus* | 2016 | Shrimp | Unknown |
| 16-B3PA-2394 | *V. parahaemolyticus* | 2016 | Shrimp | Unknown |
| 16-B3PA-2447 | *V. parahaemolyticus* | 2016 | Shrimp | Unknown |
| 16-B3PA-2472 | *V. parahaemolyticus* | 2016 | Shrimp | Ecuador |
| 16-B3PA-2473 | *V. parahaemolyticus* | 2016 | Shrimp | India |
| 16-B3PA-2516 | *V. parahaemolyticus* | 2016 | Shrimp | Unknown |
| 16-B3PA-2613 | *V. parahaemolyticus* | 2016 | Shrimp | Ecuador |
| 16-B3PA-2614 | *V. parahaemolyticus* | 2016 | Shrimp | Ecuador |
| 16-B3PA-2725 | *V. parahaemolyticus* | 2016 | Shrimp | Unknown |
| 16-B3PA-2731 | *V. parahaemolyticus* | 2016 | Shrimp | Ecuador |
| 16-B3PA-2732 | *V. parahaemolyticus* | 2016 | Shrimp | Ecuador |
| 16-B3PA-2733 | *V. parahaemolyticus* | 2016 | Shrimp | Ecuador |
| 16-B3PA-2734 | *V. parahaemolyticus* | 2016 | Shrimp | Ecuador |
| 16-B3PA-2735 | *V. parahaemolyticus* | 2016 | Shrimp | Ecuador |
| 16-B3PA-2736 | *V. parahaemolyticus* | 2016 | Shrimp | Ecuador |
| 16-B3PA-2737 | *V. parahaemolyticus* | 2016 | Shrimp | Ecuador |
| 16-B3PA-2738 | *V. parahaemolyticus* | 2016 | Shrimp | Ecuador |
| 16-B3PA-2797 | *V. parahaemolyticus* | 2016 | Shrimp | Unknown |
| 16-B3PA-2798 | *V. parahaemolyticus* | 2016 | Shrimp | Unknown |
| 16-B3PA-2826 | *V. parahaemolyticus* | 2016 | Shrimp | Unknown |
| 16-B3PA-2865 | *V. parahaemolyticus* | 2016 | Shrimp | Unknown |
| 16-B3PA-2866 | *V. parahaemolyticus* | 2016 | Shrimp | Unknown |
| 16-B3PA-2869 | *V. parahaemolyticus* | 2016 | Shrimp | Madagascar |
| 16-B3PA-2870 | *V. parahaemolyticus* | 2016 | Shrimp | Madagascar |
| 16-B3PA-2903 | *V. parahaemolyticus* | 2016 | Shrimp | Unknown |
| 16-B3PA-2904 | *V. parahaemolyticus* | 2016 | Shrimp | Unknown |
| 16-B3PA-2905 | *V. parahaemolyticus* | 2016 | Shrimp | Unknown |
| 16-B3PA-2906 | *V. parahaemolyticus* | 2016 | Shrimp | Unknown |
| 16-B3PA-3093 | *V. parahaemolyticus* | 2016 | Shrimp | Unknown |
| 16-B3PA-3094 | *V. parahaemolyticus* | 2016 | Shrimp | Unknown |
| 16-B3PA-3095 | *V. parahaemolyticus* | 2016 | Shrimp | Unknown |
| 16-B3PA-3099 | *V. parahaemolyticus* | 2016 | Shrimp | Unknown |
| 16-B3PA-3248 | *V. parahaemolyticus* | 2016 | Shrimp | Unknown |
| 16-B3PA-3249 | *V. parahaemolyticus* | 2016 | Shrimp | Unknown |
| 16-B3PA-3250 | *V. parahaemolyticus* | 2016 | Shrimp | Unknown |
| 16-B3PA-3252 | *V. parahaemolyticus* | 2016 | Shrimp | Unknown |
| 16-B3PA-3286 | *V. parahaemolyticus* | 2016 | Shrimp | Unknown |
| 16-B3PA-3341 | *V. parahaemolyticus* | 2016 | Shrimp | Unknown |
| 16-B3PA-3435 | *V. parahaemolyticus* | 2016 | Shrimp | India |
| 16-B3PA-3511 | *V. parahaemolyticus* | 2016 | Shrimp | Unknown |
| 16-B3PA-3512 | *V. parahaemolyticus* | 2016 | Shrimp | Unknown |
| 16-B3PA-3513 | *V. parahaemolyticus* | 2016 | Shrimp | Unknown |
| 16-B3PA-3515 | *V. parahaemolyticus* | 2016 | Shrimp | Ecuador |
| 16-B3PA-3518 | *V. parahaemolyticus* | 2016 | Shrimp | Ecuador |
| 16-B3PA-3519 | *V. parahaemolyticus* | 2016 | Shrimp | Unknown |
| 16-B3PA-3543 | *V. parahaemolyticus* | 2016 | Shrimp | Unknown |
| 16-B3PA-3558 | *V. parahaemolyticus* | 2016 | Shrimp | Unknown |
| 16-B3PA-3584 | *V. parahaemolyticus* | 2016 | Shrimp | Peru |
| 16-B3PA-3585 | *V. parahaemolyticus* | 2016 | Shrimp | Ecuador |
| 16-B3PA-3713 | *V. parahaemolyticus* | 2016 | Shrimp | Ecuador |
| 16-B3PA-3718 | *V. parahaemolyticus* | 2016 | Shrimp | Unknown |
| 16-B3PA-3776 | *V. parahaemolyticus* | 2016 | Shrimp | India |
| 16-B3PA-3787 | *V. parahaemolyticus* | 2016 | Shrimp | Argentina |
| 16-B3PA-3830 | *V. parahaemolyticus* | 2016 | Shrimp | Ecuador |
| 16-B3PA-3835 | *V. parahaemolyticus* | 2016 | Shrimp | Unknown |
| 16-B3PA-3846 | *V. parahaemolyticus* | 2016 | Shrimp | Unknown |
| 16-B3PA-3847 | *V. parahaemolyticus* | 2016 | Shrimp | Unknown |
| 16-B3PA-3881 | *V. parahaemolyticus* | 2016 | Shrimp | Unknown |
| 16-B3PA-3882 | *V. parahaemolyticus* | 2016 | Shrimp | Ecuador |
| 16-B3PA-3924 | *V. parahaemolyticus* | 2016 | Shrimp | Ecuador |
| 16-B3PA-3925 | *V. parahaemolyticus* | 2016 | Shrimp | Ecuador |
| 16-B3PA-3926 | *V. parahaemolyticus* | 2016 | Shrimp | Ecuador |
| 16-B3PA-3966 | *V. parahaemolyticus* | 2016 | Shrimp | Ecuador |
| 16-B3PA-4024 | *V. parahaemolyticus* | 2016 | Shrimp | Ecuador |
| 16-B3PA-4051 | *V. parahaemolyticus* | 2016 | Shrimp | Unknown |
| 16-B3PA-4091 | *V. parahaemolyticus* | 2016 | Shrimp | Ecuador |
| 16-B3PA-4092 | *V. parahaemolyticus* | 2016 | Shrimp | Ecuador |
| 16-B3PA-4475 | *V. parahaemolyticus* | 2016 | Shrimp | Unknown |
| 16-B3PA-4476 | *V. parahaemolyticus* | 2016 | Shrimp | India |
| 16-B3PA-4541 | *V. parahaemolyticus* | 2016 | Shrimp | Peru |
| 16-B3PA-4598 | *V. parahaemolyticus* | 2016 | Shrimp | Unknown |
| 16-B3PA-4615 | *V. parahaemolyticus* | 2016 | Shrimp | Unknown |
| 16-B3PA-4745 | *V. parahaemolyticus* | 2016 | Shrimp | Unknown |
| 16-B3PA-4746 | *V. parahaemolyticus* | 2016 | Shrimp | Unknown |
| 16-B3PA-4747 | *V. parahaemolyticus* | 2016 | Shrimp | Unknown |
| 16-B3PA-4830 | *V. parahaemolyticus* | 2016 | Shrimp | Nigeria |
| 16-B3PA-4869 | *V. parahaemolyticus* | 2016 | Shrimp | Unknown |
